# Poster Session II A332 ARTIFICIAL INTELLIGENCE MODELS DEMONSTRATE PROMISING BUT SUBOPTIMAL PERFORMANCE IN DIAGNOSING AND TREATING DISORDERS OF GUT-BRAIN INTERACTION

**DOI:** 10.1093/jcag/gwaf042.332

**Published:** 2026-02-13

**Authors:** M Ahn, C Parker

**Affiliations:** University of Toronto Temerty Faculty of Medicine, Toronto, ON, Canada; University Health Network, Toronto, ON, Canada

## Abstract

**Background:**

Diagnosing disorders of gut-brain interaction (DGBIs) remains a persistent challenge in gastroenterology and primary care. Large language artificial intelligence models (LLM) have emerged as a potential tool to support clinical decision-making, yet their clinical applicability remains unclear.

**Aims:**

To compare the diagnostic accuracy and treatment recommendations generated from five LLMs using clinical scenarios from the Rome IV Multidimensional Clinical Profile (MDCP).

**Methods:**

68 case scenarios representing various DGBIs were entered into Chat GPT 4.0, Google Gemini 2.5 Pro, Microsoft Copilot, OpenEvidence, and Perplexity. Each model was provided a standardized prompt to produce a diagnosis and treatment options based on the case scenario. Responses were evaluated against MDCP standards and expert consensus for diagnostic and treatment accuracy. For diagnostic accuracy, a response was correct if it matched the MDCP diagnosis or used an accepted historical term. Partially correct responses identified a clinical modifier but an incorrect MDCP diagnosis or only a part of the MDCP diagnosis. Incorrect responses did not match the MDCP diagnosis, identified an incorrect subtype of a correct diagnosis or included unrelated additional diagnoses.

**Results:**

Diagnostic accuracy was highest with Perplexity (74%), ChatGPT (72%), Google Gemini (72%), and lowest with Microsoft Copilot (65%) and OpenEvidence (65%). Regarding treatment options, ChatGPT, Google Gemini and Microsoft Copilot each generated appropriate treatment options in between 53 to 54% of cases, compared with 41% each for OpenEvidence and Perplexity. When a subgroup analysis was conducted based on the MDCP diagnostic category, LLMs had the highest accuracy diagnosing child/adolescent conditions or esophageal disorders and poorest accuracy diagnosing anorectal disorders. For treatment options, LLMs had the highest accuracy for neonate/toddler disorders and gallbladder/sphincter of Oddi disorders and the poorest accuracy for gastroduodenal disorders. Statistical analysis revealed no significant differences between LLMs for diagnostic (χ^2^(4) = 4.26, *p* = 0.372) or treatment accuracy (χ^2^(4) = 8.069, *p* = 0.089).

**Conclusions:**

LLMs demonstrated moderately accurate performance in determining the diagnosis of and treatment options for DGBIs. Diagnostic errors and omission of several options in the treatment of DGBIs highlights the risk of premature clinical adoption and need for further validation on strategies for clinical integration.

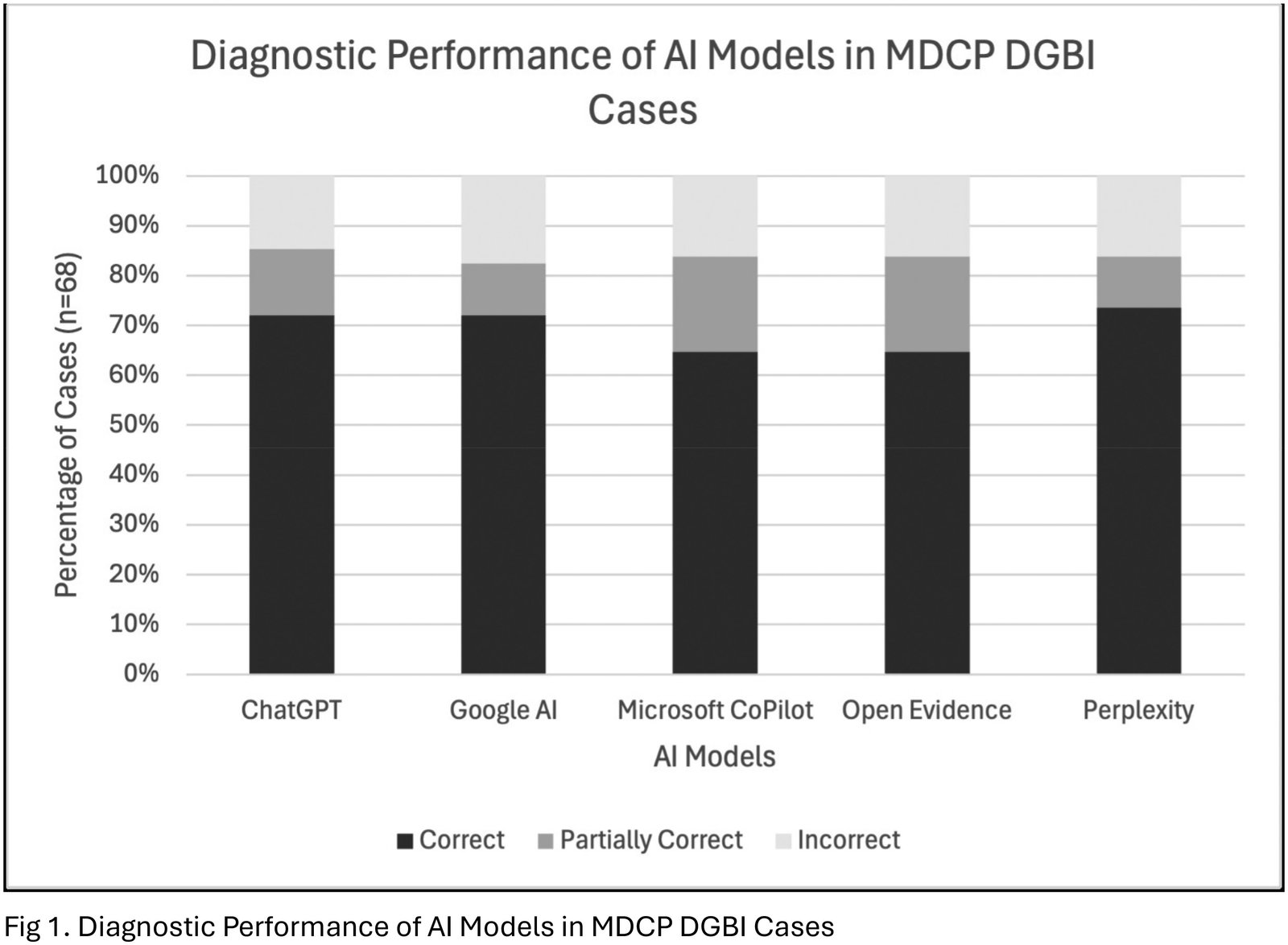

**Funding Agencies:**

None

